# Impacts of primary tumor location on outcomes in patients undergoing hepatectomy for colorectal liver metastasis vary according to tumor burden

**DOI:** 10.3389/fsurg.2022.992991

**Published:** 2022-11-04

**Authors:** Hong-Wei Wang, Li-Jun Wang, Juan Li, Kun Wang, Bao-Cai Xing

**Affiliations:** Hepatopancreatobiliary Surgery Department I, Key Laboratory of Carcinogenesis and Translational Research, Ministry of Education, Peking University School of Oncology, Beijing Cancer Hospital and Institute, Beijing, China

**Keywords:** colorectal liver metastasis, tumor burden score, primary tumor location, survival analysis, hepatectomy

## Abstract

**Purpose:**

The purpose of this study was to verify whether the prognostic value of primary tumor location (PTL) for patients undergoing resection for colorectal liver metastasis (CRLM) is affected by tumor burden.

**Methods:**

Patients who underwent a first curative-intent surgery for CRLM from 2006 to 2017 were enrolled. The imaging tumor burden score (TBS) was calculated as TBS^2^ = (maximum tumor diameter in cm)^2^ + (number of lesions)^2^. Then, the prognostic role of PTL was assessed in different TBS zones.

**Results:**

The patient population consisted of 524 left-sided (LS) and 118 right-sided (RS) primary tumors. The distribution of TBS in the patient cohort was: Zone1: TBS <3 [*n* = 161 (25.1%)], zone 2: TBS ≥3 to <7 [*n* = 343 (53.4%)], and zone 3: TBS ≥7 [*n* = 138 (21.5%)]. In the whole cohort, the 5-year overall survival (OS) in the RS group was worse than that in the LS group (35.6% vs. 45.4%). However, after adjustment for known prognostic confounders, the RS group was not independently associated with a poorer OS (HR 1.18, *p* = 0.247). Among patients with TBS <7, OS in the RS group was significantly shorter than that in the LS group in both univariate and multivariate analyses. The prognostic role of PTL remained significant after propensity score matching or excluding patients who received anti-EGFR agents. Conversely, the association between PTL and OS was no longer evident in patients with TBS ≥7.

**Conclusion:**

The current study demonstrates that the prognostic value of PTL varies by TBS, and RS tumors are only associated with shorter survival in patients with low or medium TBS.

## Introduction

Colorectal cancer (CRC) is common in China and ranks fifth in cancer-related death (1). The liver is the most common site of CRC metastasis. Surgical resection has been proven to be the best strategy for patients with colorectal cancer liver metastasis (CRLM) (2). In recent years, advances in therapeutic procedures, such as intensified chemotherapy regimens (for instance, FOLFOXIRI plus biological agents) and one-stage ultrasound-guided parenchymal preserving resections, have expanded the indications for resection of CRLM (3–5). Unfortunately, the majority of patients experience tumor recurrence, and their 10-year survival rate is still ≤30% (6, 7). Therefore, the identification of patients likely to have poor prognosis is essential for successful personalized treatments.

Primary tumor location (PTL) has been identified as a prognostic factor in patients with advanced metastatic CRC (8). Growing evidence supports the view that right-sidedness (RS) was associated with worse prognosis when compared with left-sidedness (LS) in patients with unresectable mCRC, with possibly no benefit from anti-EGFR agents (9–11). For resectable CRLM, two recent meta-analyses demonstrated that LS is a substantially better prognostic factor in terms of overall survival (12, 13). These authors suggested that the prognostic role of PTL is also valid for surgically resected CRLM (12, 13). Nevertheless, CRLM is a complex and heterogeneous disease, and a recent study by the International Genetic Consortium for CRLM concluded that RS is a good predictor of overall survival (OS) only in patients with K-RAS wild-type tumors (14). Hence, PTL may have different influences on the prognosis of CRLM under diverse scenarios.

The “tumor burden score” (TBS) was first proposed by Sasaki et al. (15, 16) for CRLM, and the prognostic value of TBS is superior to that of tumor size and traditional clinical risk scores. Previous studies have demonstrated that the prognostic value of margin status changes at different levels of TBS (17, 18). As such, it was hypothesized that the prognostic value of PTL may differ in resectable CRLM with different TBS values, and the purpose of this study was to test this hypothesis.

## Patients and methods

### Cohort selection and data collection

Patients undergoing a first liver resection for CRLM at the Hepato-Pancreato-Biliary Surgery Department I, Peking University Cancer Hospital, in 2006 and 2017 were consecutively enrolled in this study. Only patients who underwent complete resection of both primary and CRLM were included in this study. Patients were excluded if operative mortality occurred and patients lost to follow-up. The clinical information for each patient, including sex, age, primary tumor characteristics (location, depth of invasion, presence/absence of lymph node metastases), preoperative factors [administration of preoperative chemotherapeutic agents, preoperative levels of carcinoembryonic antigen (CEA), and synchronous vs. metachronous liver metastasis], CRLM characteristics (maximum tumor size, number of tumors, presence of extrahepatic disease, margin status, status of RAS/BRAF mutations), perioperative outcomes (blood loss, blood transfusion, and major vs. minor resection), postoperative therapy and follow-up data, was obtained from a prospective hepatic resection database and supplemented by medical record reviews. The present study was approved by the Institutional Review Board of the hospital.

### Definition and patient management

The distal third of the transverse colon was defined as the boundary between the right and left colon by embryological origin (19). However, this definition was difficult to apply in retrospective assessments of clinical databases. Thus, the splenic flexure was used in this study to differentiate between RS and LS tumors (20–23). Synchronous metastasis was defined as liver metastasis detected upon or before diagnosis of the primary tumor. R1 resection was defined as the presence of tumor cells within 1 mm from the surgical margin. Hepatic resection including 3 or more liver segments was used to define a major resection. Lost to follow-up was defined as ≥12 months without visit, equivalent to missing at least 2 consecutive visits. In our study, 11 cases were lost to follow-up and the lost rate was 1.6%.

All patients underwent routine laboratory evaluation and preoperative imaging to assess their extrahepatic disease and resectability of CRLM. Preoperative staging included contrast-enhanced CT, MRI scan or positron emission tomography/CT. Resectability was defined as macroscopic complete resection could be achieved with liver remnant volume equal to at least 30% or 40% (for patients with chemotherapy-induced liver injury) of the total estimated liver volume. Preoperative fluorouracil-based chemotherapy was recommended unless single, metachronous resectable metastases were present or the patient refused. Restaging was performed every 1–2 months during chemotherapy. Hepatectomy was performed at 4–6 weeks after chemotherapy. Portal vein embolization or ligation with two-stage hepatectomy was performed to achieve an sufficient remnant liver volume. CRLM or primary tumor samples were analyzed for RAS and BRAF mutations using techniques described previously (20).

During the surgery, an intraoperative ultrasound scan was routinely used to assess lesions and vascular anatomy and to detect new metastases. An intermittent Pringle maneuver was also applied when deemed necessary by the surgeon. Combined resection/radiofrequency ablation (RFA) was selected when not all tumors could be removed by single hepatectomy or if the tumors were deeply embedded in the remnant liver. Routine 6-month systemic therapy was given after the surgery along with necessary adjuvant chemotherapy. All subjects were followed up every 3 months for the first 2 years and then every 6 months in subsequent years. At each follow-up, CEA measurement, liver function tests, and imaging studies, such as thoracic, pelvic and abdominal enhanced computed tomography or abdominal MRI scans, were performed to detect recurrence.

### Statistical analysis

TBS was calculated using the formula below: TBS^2^ = (maximum tumor diameter in cm)^2^ + (number of tumors)^2^ (15). The size and number of tumors were calculated from preoperative contrast-enhanced CT or MRI scans of the abdomen. The exact numbers and percentages were used to express categorical data; medians with interquartile range (IQR) were used to display continuous data. Chi square or Fisher's exact tests were employed to compare categorical variables, and the Mann–Whitney *U* test was used to compare continuous variables between groups. OS was defined as the time from hepatectomy to death or the last follow-up and was calculated by Kaplan–Meier analysis; differences in estimated OS between groups were compared using the log-rank test. A Cox proportional hazards model was used to assess the correlation of clinicopathologic and genetic variables with OS. Variables that were identified in univariate analysis (*p* < 0.10) were retained in the multivariable model, and the hazard ratio (HR) and 95% confidence interval were calculated for the strength of association between OS and each variable. A 2-sided *p* value <0.05 was considered statistically significant. Finally, nearest neighbor propensity score matching (PSM) was conducted to reduce selection biases. Confounders were selected as variables (RAS status, gender and whether received preoperative chemotherapy) inconsistently distributed between RS and LS cancers in patients with TBS <7 (*p* < 0.10). PSM analysis was performed using the MatchIt package in R 3.5.1 with the caliper set to 0.05. SPSS Statistics 23.0 (SPSS Inc., Chicago, IL) and R 3.5.1. (https://cran.r-project.org/) were used for the above analyses.

## Results

### Clinicopathologic characteristics of the study cohort

During the study period, 699 patients underwent 766 CRLM resections. Of these patients, 57 were excluded from the study population for the following reasons: 4 patients experienced postoperative mortality, 22 patients had incomplete resection, 20 patients underwent repeated resection, and 11 patients were lost to follow-up. Of the remaining 642 patients, 524 had LS (81.6%), 118 had RS (18.4%) primary tumors, and the status of RAS/BRAF was available in 634 (98.8%). Clinicopathological characteristics stratified by PTL are summarized in Table 1. In brief, patients with LS primary tumors were primarily characterized by male sex (*p* = 0.000), a higher rate of preoperative chemotherapy (*p* = 0.017) and a lower chance of RAS/BRAF mutations (*p* = 0.000). Other clinicopathologic features were similar between the two groups.

**Table 1:** Clinical, pathological and molecular characteristics stratified according to PTL (*N* = 642).

	Left-sided (*n* = 524)	Right-sided (*n* = 118)	*p*
Patient characteristics			
Age, years, median (IQR)	58.0 (49.0–63.0)	59 (52.0–65.0)	0.145
Gender (%)			
Male	360 (68.7)	58 (49.2)	0.000
Primary tumor characteristics			
T stage (%)			
T1 or T2 stage	47 (8.9)	7 (3.7)	0.360
T3 or T4 stage	477 (91.1)	111 (96.3)	
Node-positive (%)	369 (70.4)	79 (66.9)	0.506
Preoperative factors			
Preoperative chemotherapy (%)	367 (70.0)	69 (58.4)	0.017
Anti-EGFR (%)	84 (22.9)	13 (18.8)	0.201
Bevacizumab (%)	99 (27.0)	18 (26.1)	0.429
CEA, median (IQR)	6.66 (3.26–21.76)	7.87 (3.87–26.87)	0.282
Synchronous metastasis (%)	240 (44.3)	49 (41.5)	0.414
CRLM characteristics			
TBS, median (IQR)	4.44 (3.11–6.38)	4.38 (2.86–6.58)	0.927
Bilateral disease (%)	249 (47.5)	54 (45.8)	0.289
Ras mutation (*n* = 634) (%)^a^	171 (33.1)	60 (51.3)	0.000
Extrahepatic disease (%)	72 (13.7)	20 (16.9)	0.369
Perioperative outcomes			
Blood loss (ml), median (IQR)	200 (100–300)	175 (100–200)	0.272
Red blood cell transfusion (%)	48 (9.2)	17 (14.4)	0.093
Major hepatectomy (%)	92 (17.6)	18 (15.3)	0.591
Concurrent RFA (%)	52 (9.9)	10 (8.5)	0.732
R1 resection (%)	158 (30.2)	25 (25.5)	0.201
Adjuvant chemotherapy (*n* = 626) (%)^a^	372 (72.9)	85 (75.2)	0.587

CEA, carcinoembryonic antigen; TBS, tumor burden score; CI, confidence interval.

^a^ The number of patients with available data.

### Long-term outcomes in the entire cohort and the subanalysis of OS in patients with different RAS statuses

**Figure 1:**
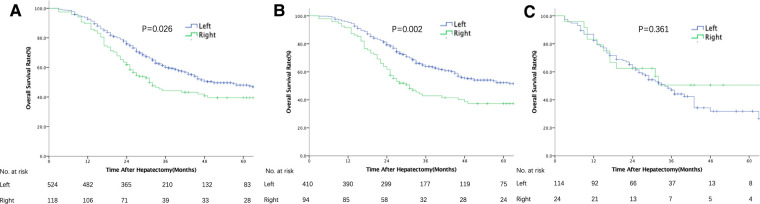
Overall survival after resection of colorectal liver metastases stratified by primary tumor location (left vs. right). (**A**) In the entire cohort. (**B**) In patients with TBS <7. (**C**) In patients with TBS ≥7.

The median follow-up was 31 months. The median OS of all patients was 47 months (36.6–57.4), and 1-, 3-, and 5-year survival rates were 92%, 57.2%, and 46.6%, respectively. In univariate analysis, patients with RS tumors had a markedly lower OS than those with LS tumors, with 3- and 5-year OS rates of RS and LS tumor patients of 44.7% vs. 59.3% and 35.6% vs. 45.4%, respectively (*p* = 0.026, Figure 1). However, after adjustment for known prognostic confounders, RS was not independently associated with a worse OS (HR 1.18, 95% confidence interval [CI] 0.89–1.54, *p* = 0.247). On the other hand, the lymph node status of the primary tumor (HR1.48, 95% CI 1.13–1.92, *p* = 0.004), increasing TBS (HR1.06, 95% CI 1.03–1.09, *p* = 0.000), extrahepatic metastasis (EHD) (HR1.99, 95% CI 1.51–2.64, *p* = 0.000) and the presence of RAS/BRAF mutations (HR2.00, 95% CI 1.59–2.52, *p* = 0.000) were all independently associated with a worse OS in multivariate analysis (Table 2). To verify the results from a previous study, survival analysis was performed in patients with different RAS statuses. Unfortunately, RS was not associated with OS after liver resection of CRLM in this cohort, regardless of RAS status (wild-type: LS vs. RS, median OS 77 vs. 49 months, *p* = 0.317; mutated: LS vs. RS, median OS 33 vs. 24 months, *p* = 0.359, Supplementary Figure S1).

**Table 2:** Univariate and multivariate analyses for the overall survival in the entire cohort.

	Univariate		Multivariate	
	HR (95% CI)	*p*	HR (95% CI)	*p*
Patient age >60	0.88 (0.71–1.08)	0.221	-	
Female gender	0.87 (0.70–1.08)	0.189	-	
Primary tumor location				
Left-sided primary	Ref		Ref	
Right-sided primary	1.32 (1.02–1.70)	0.033	1.18 (0.89–1.54)	0.247
Primary tumor stage				
T1 and T2	Ref			
T3 and T4	1.05 (0.92–1.20)	0.489	-	
Lymph node metastasis	1.43 (1.13–1.81)	0.003	1.48 (1.13–1.92)	0.004
Preoperative chemotherapy	0.93 (0.74–1.16)	0.498		
CEA >20 ng/dl	1.13 (0.89–1.42)	0.304	-	
Synchronous liver metastases	0.88 (0.71–1.08)	0.211	-	
TBS (continuous variable)	1.09 (1.05–1.12)	0.000	1.06 (1.03–1.09)	0.000
Bilateral liver disease	1.20 (0.96–1.50)	0.015	-	
RAS/BRAF status				
Wild-type tumors	Ref		Ref	
Mutated	2.06 (1.64–2.58)	0.000	2.00 (1.59–2.52)	0.000
Extrahepatic disease	2.08 (1.57–2.74)	0.000	1.99 (1.51–2.64)	0.000
Red blood cell transfusion	1.37 (0.98–1.91)	0.068	1.25 (0.89–1.76)	0.205
Intraoperative ablation	0.85 (0.54–1.34)	0.474		
R1 resection	1.14 (0.92–1.42)	0.233		
Adjuvant chemotherapy	0.82 (0.64–1.05)	0.116		

CEA, carcinoembryonic antigen; TBS, tumor burden score; CI, confidence interval.

### Prognostic implication of PTL at different levels of TBS

The patients in this cohort were divided into 3 groups according to their TBS (13): zone 1: TBS <3, *n* = 161 (25.1%); zone 2: TBS ≥3 to <7, *n* = 343 (53.4%); and zone 3: TBS ≥7, *n* = 138 (21.5%). Among patients with high TBS (zone 3), OS did not differ between the RS and LS groups in both univariate and multivariate analyses (Figure 1 and Supplementary Table S1). In contrast, among patients with a medium or low TBS (zones 1 and 2), OS after liver resection of CRLM was shorter among patients with a RS primary tumor (median: 31 months, 5-year OS: 37.3%) than that in patients with a LS tumor (median: 65 months, 5-year OS: 52.2% *p* = 0.002, Figure 1). In multivariate analysis and after adjusting for other competing risk factors, PTL remained an independent factor associated with a worse OS (HR 1.41; 95% CI 1.04–1.91, *p* = 0.027) (Table 3). We also evaluated the patterns of recurrence patterns and salvage treatments (resection, ablation and/or stereotactic body radiotherapy of all recurrent disease). In our cohort, the PTL did not impact recurrence patterns. TBS >7 was associated with a significantly higher cumulative incidence of intrahepatic recurrence. The salvage treatment and the incidence of lung and other site recurrence did not vary by TBS (Supplementary Tables S2, S3).

**Table 3:** Univariate and multivariate survival analyses in patients of TBS <7.

	Univariate		Multivariate	
	HR (95% CI)	*p*	HR (95% CI)	*p*
Patient age >60	0.89 (0.69–1.16)	0.404	-	
Female gender	0.87 (0.67–1.14)	0.311	-	
Primary tumor location				
Left-sided primary	Ref		Ref	
Right-sided primary	1.61 (1.19–2.17)	0.002	1.41 (1.04–1.91)	0.027
Primary tumor stage				
T1 and T2	Ref			
T3 and T4	1.06 (0.92–1.22)	0.413	-	
Lymph node metastasis	1.58 (1.17–2.12)	0.003	1.65 (1.23–2.22)	0.001
Preoperative chemotherapy	1.11 (0.85–1.44)	0.452		
CEA >20 ng/dl	1.03 (0.77–1.38)	0.846	-	
Synchronous liver metastases	0.94 (0.73–1.22)	0.637	-	
TBS (continuous variable)	1.13 (1.03–1.24)	0.007	1.13 (1.03–1.23)	0.009
Bilateral liver disease	0.95 (0.73–1.24)	0.694	-	
RAS/BRAF status				
Wild-type tumors	Ref		Ref	
Mutated	1.98 (1.52–2.57)	0.000	1.89 (1.45–2.46)	0.000
Extrahepatic disease	2.04 (1.46–2.83)	0.000	2.00 (1.43–2.79)	0.000
Red blood cell transfusion	1.24 (0.81–1.89)	0.330		
Intraoperative ablation	0.68 (0.34–1.38)	0.286		
R1 resection	1.16 (0.90–1.49)	0.254		
Adjuvant chemotherapy	0.85 (0.64–1.14)	0.281		

CEA, carcinoembryonic antigen; TBS, tumor burden score; CI, confidence interval.

### Subanalysis of the prognostic value of PTL for OS after PSM or excluding use of anti-EGFR agents in patients with <TBS7

Clinicopathological and genetic characteristics were compared and stratified by PTL in patients with TBS <7 (Table 4). To adjust for possible risk factors that differ between RS and LS CRLM, a propensity score was evaluated. Multivariate analysis demonstrated that PTL (HR 2.71; 95% CI 1.74–4.21, *p* = 0.000), EHD (HR 2.02; 95% CI 1.18–3.47, *p* = 0.002) and RAS/BRAF mutations (HR 2.02; 95% CI 1.30–3.13, *p* = 0.001) were independent risk factors for OS (Table 5). Additional investigations were carried out by excluding patients who received anti-EGFR agents, and PTL was still a poor prognostic factor for OS in multivariate analysis (Supplementary Table S4).

**Table 4:** Clinical, pathological and molecular characteristics stratified according to PTL in patients of TBS <7 (*N* = 504).

	Left-sided (*n* = 410)	Right-sided (*n* = 94)	*p*
Patient characteristics			
Age, years, median (IQR)	59.0 (50.0–65.0)	59.0 (51.0–65.0)	0.817
Gender (%)			
Male	272 (65.9)	46 (48.9)	0.002
Primary tumor characteristics			
T stage (%)			
T1 or T2 stage	43 (10.5)	5 (5.3)	0.179
T3 or T4 stage	367 (89.5)	89 (94.7)	
Node-positive (%)	281 (68.5)	64 (68.1)	0.932
Preoperative factors			
Preoperative chemotherapy (%)	272 (66.3)	51 (54.3)	0.028
Anti-EGFR (%)	53 (12.9)	9 (9.5)	0.372
Bevacizumab (%)	65 (15.9)	11 (11.7)	0.310
CEA, median (IQR)	20.74 (11.12–44.47)	22.6 (12.09–43.74)	0.825
Synchronous metastasis (%)	203 (49.5)	44 (46.8)	0.636
CRLM characteristics			
TBS, median (IQR)	3.64 (2.69–5.01)	3.69 (2.69–5.12)	0.789
Bilateral disease (%)	161 (39.3)	37 (39.3)	0.987
Ras mutation (*n* = 499) (%)^a^	131 (32.0)	49 (52.1)	0.000
Extrahepatic disease (%)	51 (12.4)	16 (17.0)	0.238
Perioperative outcomes			
Blood loss (ml), median (IQR)	100 (100–300)	100 (100–200)	0.670
Red blood cell transfusion (%)	32 (7.8)	12 (12.8)	0.124
Major hepatectomy (%)	62 (15.1)	14 (14.9)	0.956
Concurrent RFA (%)	27 (5.9)	4 (4.3)	0.394
R1 resection (%)	105 (25.6)	20 (21.3)	0.380
Adjuvant chemotherapy (*n* = 490) (%)^a^	291 (72.6)	64 (71.9)	0.900

CEA, carcinoembryonic antigen; TBS, tumor burden score; CI, confidence interval.

^a^ The number of patients with available data.

**Table 5:** Univariate and multivariate survival analyses after PSM in patients of TBS <7.

	Univariate		Multivariate	
	HR (95% CI)	*p*	HR (95% CI)	*p*
Patient age >60	0.97 (0.64–1.48)	0.894		
Female gender	0.87 (0.57–1.32)	0.514	-	
Primary tumor location				
Left-sided primary	Ref		Ref	
Right-sided primary	2.64 (1.70–4.01)	0.000	2.71 (1.74–4.21)	0.000
Primary tumor stage				
T1 and T2	Ref			
T3 and T4	1.36 (0.43–4.29)	0.604	-	
Lymph node metastasis	1.32 (0.84–2.071)	0.235		
Preoperative chemotherapy	0.97 (0.64–1.47)	0.887		
CEA >20 ng/dl	1.12 (0.70–1.80)	0.628	-	
Synchronous liver metastases	0.76 (0.50–1.15)	0.192	-	
TBS (continuous variable)	1.05 (0.91–1.22)	0.517		
Bilateral liver disease	0.97 (0.63–1.49)	0.876	-	
RAS/BRAF status				
Wild-type tumors	Ref		Ref	
Mutated	2.07 (1.34–3.19)	0.001	2.02 (1.30–3.13)	0.001
Extrahepatic disease	2.44 (1.43–4.16)	0.011	2.02 (1.18–3.47)	0.002
Red blood cell transfusion	1.40 (0.80–2.40)	0.242		
Intraoperative ablation	0.29 (0.01–1.99)	0.203		
R1 resection	0.83 (0.54–1.28)	0.389		
Adjuvant chemotherapy	1.04 (0.65–1.66)	0.860		

CEA, carcinoembryonic antigen; TBS, tumor burden score; CI, confidence interval.

## Discussion

In this retrospective study, the impact of PTL on OS was analyzed in a single cohort of 642 CRLM patients who underwent complete resection of both primary and metastatic tumors. PTL was demonstrated to be an independent prognostic factor for patients with low and medium tumor burdens. However, among patients with a high tumor burden, RS was not independently associated with worse OS. Additionally, tumor laterality still influenced OS when confounders were balanced with PSM or when patients receiving anti-EGFR agents or with EHD were excluded from the analysis.

The right and left sides of CRC have unequal etiologies, including different embryologic origins, different carcinogenesis mechanisms, and different microbiota distributions (24, 25). In recent years, PTL has been confirmed as a principle prognostic factor in metastatic CRC. Compared to RS metastatic CRC, LS patients had a significantly lower chance of progression and longer OS after receiving palliative chemotherapy, and the adverse prognostic role of RS tumors in resectable CRLM has also been reported in two meta-analyses (12, 13). Although the two meta-analyses found a prognostic role for PTL in terms of OS, approximately half of the enrolled studies [5/12, 22/43] did not show a better OS for LS CRLM, making the results paradoxical. The intricate and heterogeneous tumor burden and biology of CRLM may explain this inconsistency. In a recent study, Margonis et al. (14) used tumor markers to minimize tumor heterogeneity, and it was found that the prognostic role of PTL was affected by K-RAS status. However, the above findings were not validated in the present study, even though the life expectancy of LS was almost 1.5 times that of RS in patients with wild-type RAS/BRAF (median OS, 77 vs. 49 months). The race discrepancy and low number of RS patients participating in this study may be responsible for the opposite outcomes. Thus, other possible factors were studied to adjust for confounders in this cohort.

TBS was derived from a “Metro-ticket” prognostic model (26) of hepatocellular carcinoma (HCC) and was first applied in CRLM by Sasaki et al. TBS can precisely estimate tumor burden by integrating tumor size, and the number of CRLMs and imaging TBSs have a strong correlation with pathologic TBS. TBS was selected to stratify patients in two studies to investigate whether the prognostic role of resection margin status changes (17, 18). Both studies found a reduced effect of lost margin status on OS when TBS increased. Therefore, this score was chosen in this study to examine whether the prognostic association of PTL in resectable CRLM also changes in different TBS groups. Interestingly, the data confirmed the prognostic role of PTL only in patients with low or medium TBS, whereas the association of RS with poorer long-term outcomes became less significant among patients with high TBS. There were more patients in the cohort of this study in TBS group 3 than in previous studies involving a Japanese cohort and a Baltimore cohort (17). Consequently, the various degrees of TBS in different cohorts might account for why the prognostic association of PTL was confirmed in some studies but not in patients undergoing resection of CRLM and the long-term prognostic role of PTL was more significant in non-Asian populations than in the Asian population. Oshi et al. (17) inferred that higher TBS may represent higher malignancy. The major determinant of tumor aggressiveness influences the prognosis of these patients. The results of this study were consistent with the roles of RAS status and EHD, the two pivotal variables representing poor tumor behaviors that were confirmed as independent predictors of inferior OS among patients with high TBS. Thus, tumor sidedness should not be used as a decisive factor to exclude hepatectomy in patient populations with a high tumor burden.

The “Metro-ticket” concept was derived from HCC patients and may also be extended to other scenarios. In patients with low or medium TBS, LS and RS act as two destinations, with RS having a longer trip to the central metro station (potential cure) than LS; thus, RS patients need to pay a higher price (more extensive therapy). For instance, among patients with limited disease (TBS <7), chemotherapy may be routinely recommended to RS patients, though a multicenter, randomized, and controlled phase 3 trial did not show an OS benefit with the combination of surgery and perioperative chemotherapy in the neoadjuvant setting. Moreover, intensified chemotherapy, such as triplet plus biologic therapy, should be considered for the treatment of RS tumors. Although no previous study has verified this combination regimen in resectable CRLM, retrospective analysis of the TRIBE trial by GONO suggested that FOLFOXIRI plus bevacizumab can effectively weaken the intrinsic aggressiveness of RS mCRC (27). As such, this regimen may be a preferable choice for patients with resectable RS CRLM if they meet the clinical criteria for the utilization of the triplet regimen.

This study had several limitations. First, this study was carried out at a single institution and employed a retrospective design. Thus, selection bias was inevitable. However, selection bias was minimized by controlling for possible competing factors in multivariate analysis and PSM. Second, as most of the patients received systemic therapy at other hospitals, the preoperative chemotherapy regimens were not standardized, and detailed information on response to preoperative chemotherapy was not available. Therefore, it is possible that a higher number of RS patients were nonresponders, thus interfering with our survival analysis because many previous studies have shown that a worse preoperative response is associated with shorter survival (28–30). Last, patients with rectal primary cancers were included in this study. Rectal cancer was considered to be a distinct entity from colon cancer due to different therapeutic strategies. Hence, the results should be generalized with caution. We included patients with rectal cancer based on the following reasons. First, from the perspective of embryology, the splenic flexure, descending colon and sigmoid rectum and the upper part of the anal canal all originate from the hindgut (31). Second, though rectal cancer is not typically included in most studies concerning colorectal cancer's location as a prognostic factor for survival, in the few studies that do, the results are consistent with RS was associated with worse prognosis when compared with left-sidedness LS (8, 20, 32, 33). In the future, the impact of PTL on the OS of resectable CRLM should be further investigated by well-designed prospective studies.

## Conclusion

In summary, this study indicates that the impact of RS primary tumors on OS is only relevant for low and medium tumor burden profiles. Therefore, intensified systemic therapy may be needed for these patients. Conversely, PTL was not associated with long-term survival among patients with high TBS. Tumor laterality may not be a determining factor to exclude hepatectomy in this patient population.

## Data Availability

The original contributions presented in the study are included in the article/**Supplementary Material**, further inquiries can be directed to the corresponding author/s.
