# Supplementary material for: Impacts of primary tumor location on outcomes in patients undergoing hepatectomy for colorectal liver metastasis vary according to tumor burden
Source: Front Surg. 2022 Nov 4;9:992991. doi: 10.3389/fsurg.2022.992991 (PMC9672368; doi:10.3389/fsurg.2022.992991)
Supplement: Supplementary file 1 [file Datasheet1.docx]

**SUPPLEMENTARY MATERIAL TO:**

**The Prognostic Impact of Primary Tumor Location Following Hepatectomy for Colorectal liver Metastasis varied according to the Tumor burden**

**Hong-Wei Wang, MD ^1^, Ke-Min Jin, MD ^1^, ,Juan Li ^1^, Kun Wang, MD ^1^,and Bao-Cai Xing, MD ^1^**

Hepatopancreatobiliary Surgery Department I, Key Laboratory of Carcinogenesis and Translational Research, Ministry of Education, Peking University School of Oncology, Beijing Cancer Hospital and Institute, Haidian District, Beijing, China

**Table of Contents**

**Supplementary table 1.** Univariate and multivariable survival analysis in patients TBS≥**7**…………………………………………………………………………………..2

**Supplementary table 2.** Recurrence Patterns and Subsequent therapy in CRLM patients with different TBS…………………………..………………………………………………..3

**Supplementary table 3.** Recurrence Patterns and Subsequent therapy in CRLM patients with different PTL…………………………..………………………………………………..3

**Supplementary table 4.** Univariate and multivariable survival analysis in patients TBS<7 with excluding received anti-EGFR agents……………………………………………..……….4

**Supplementary Figure 1.** Overall survival after resection of colorectal liver metastases stratified by primary tumor location(Left versus Right)……………………………………………5

**Supplementary table 1. Univariate and multivariable survival analysis in patients TBS**≥**7(N=138)**

|  | **Univariate** | | |  | | **Multivariate** | |  |
| --- | --- | --- | --- | --- | --- | --- | --- | --- |
|  | **HR (95% CI)** | ***p*** |  | | **HR (95% CI)** | | ***p*** | |
| Patient age>60 | 0.76 (0.46-1.25) | 0.280 |  | |  | |  | |
| Female gender | 0.61 (0.38-0.99) | 0.043 |  | | 0.67 (0.41-1.09) | | 0.106 | |
| Primary tumor location |  |  |  | |  | |  | |
| Left-sided primary | Ref |  |  | |  | |  | |
| Right-sided primary | 0.75 (0.39-1.42) | 0.369 |  | |  | |  | |
| Primary tumor stage |  |  |  | |  | |  | |
| T1 & T2 | Ref |  |  | |  | |  | |
| T3 &T4 | 0.59 (0.22-1.63) | 0.312 |  | | - | |  | |
| Lymph Node metastasis | 1.26 (0.74-2.14) | 0.393 |  | |  | |  | |
| Preoperative chemotherapy | 0.97 (0.55-1.73) | 0.921 |  | |  | |  | |
| CEA >20 ng/dl | 1.14 (0.71-1.84) | 0.586 |  | | - | |  | |
| Synchronous liver metastases | 0.82 (0.50-1.36) | 0.441 |  | | - | |  | |
| TBS(continuous variable ) | 1.07 (1.01-1.13) | 0.018 |  | | 1.03 (0.97-1.09) | | 0.369 | |
| Bilateral liver disease | 0.82(0.46-1.44) | 0.480 |  | | - | |  | |
| RAS/BRAF stasus  Wild-type tumors | Ref |  |  | | Ref | |  | |
| Mutated | 2.53(1.60-3.99) | 0.000 |  | | 2.49 (1.55-3.88) | | 0.000 | |
| Extrahepatic disease | 2.11 (1.25-3.56) | 0.005 |  | | 2.10 (1.23-3.58) | | 0.006 | |
| Red Blood Cell Transfusion | 1.44(0.82-2.53) | 0.210 |  | |  | |  | |
| Intraoperative ablation | 0.75 (0.40-1.39) | 0.360 |  | |  | |  | |
| R1 resection | 0.84 (0.53-1.32) | 0.442 |  | |  | |  | |
| Adjuvant chemotherapy | 0.73 (0.44-1.19) | 0.204 |  | |  | |  | |

CEA, carcinoembryonic antigen; TBS, Tumor Burden Score; CI, confidence interval.

Supplementary table 2. Recurrence Patterns and Subsequent therapy in CRLM patients with different TBS

| Variables | TBS<7 (504) | | TBS>7(138) | P |
| --- | --- | --- | --- | --- |
| Intrahepatic recurrence(%) | | 271 (53.7) | 111 (80.4) | 0.000 |
| Pulmonary metastasis(%) | | 143 (28.4) | 45 (32.6) | 0.333 |
| Other sites(%) | | 141 (28.0) | 33 (23.9) | 0.341 |
| Salvage resection(%) | | 83 (26.1) | 19 (18.3) | 0.115 |
| Salvage treatment(%) | | 178 (60.0) | 53 (50.9) | 0.366 |

Supplementary table 3. Recurrence Patterns and Subsequent therapy in CRLM patients with different PTL

| Variables | Left-sided(524) | Right-sided(118) | P |
| --- | --- | --- | --- |
| Intrahepatic recurrence(%) | 307 (58.6) | 75 (63.6) | 0.320 |
| Pulmonary metastasis(%) | 161 (30.7) | 27 (22.9) | 0.091 |
| Other sites(%) | 146 (27.9) | 28 (23.7) | 0.361 |
| Salvage resection(%) | 86 (24.9) | 16 (21.1) | 0.483 |
| Salvage treatment(%) | 202 (58.4) | 29 (38.7) | 0.002 |

**Supplementary table 4. Univariate and multivariable survival analysis in patients TBS<7 with excluding received anti-EGFR agents (N=442)**

|  | **Univariate** | | |  | | **Multivariate** | |  |
| --- | --- | --- | --- | --- | --- | --- | --- | --- |
|  | **HR (95% CI)** | ***p*** |  | | **HR (95% CI)** | | ***p*** | |
| Patient age>60 | 0.90(0.68-1.18) | 0.438 |  | |  | |  | |
| Female gender | 0.78 (0.59-1.03) | 0.079 |  | | 0.93 (0.70-1.24) | | 0.632 | |
| Primary tumor location |  |  |  | |  | |  | |
| Left-sided primary | Ref |  |  | | Ref | |  | |
| Right-sided primary | 1.67 (1.22-2.28) | 0.001 |  | | 1.48 (1.08-2.04) | | 0.015 | |
| Primary tumor stage |  |  |  | |  | |  | |
| T1 & T2 | Ref |  |  | |  | |  | |
| T3 &T4 | 1.06 (0.92-1.23) | 0.427 |  | | - | |  | |
| Lymph Node metastasis | 1.49 (1.10-2.03) | 0.011 |  | | 1.59 (1.16-2.17) | | 0.004 | |
| Preoperative chemotherapy | 1.12 (0.85 -1.48) | 0.417 |  | |  | |  | |
| CEA >20 ng/dl | 0.99 (0.73-1.37) | 0.985 |  | | - | |  | |
| Synchronous liver metastases | 0.95 (0.72-1.24) | 0.685 |  | | - | |  | |
| TBS(continuous variable ) | 1.09 (0.99-1.21) | 0.060 |  | | 1.09 (0.99-1.20) | | 0.073 | |
| Bilateral liver disease | 0.98 (0.74-1.30) | 0.883 |  | | - | |  | |
| RAS/BRAF stasus  Wild-type tumors | Ref |  |  | | Ref | |  | |
| Mutated | 1.99(1.51-2.62) | 0.000 |  | | 1.85(1.40-2.46) | | 0.000 | |
| Extrahepatic disease | 2.16 (1.54-3.03) | 0.000 |  | | 2.09(1.48-2.94) | | 0.000 | |
| Red Blood Cell Transfusion | 1.28 (0.81-2.04) | 0.293 |  | |  | |  | |
| Intraoperative ablation | 0.65 (0.31-1.38) | 0.264 |  | |  | |  | |
| R1 resection | 1.18 (0.91-1.53) | 0.231 |  | |  | |  | |
| Adjuvant chemotherapy | 0.91 (0.67-1.24) | 0.558 |  | |  | |  | |

CEA, carcinoembryonic antigen; TBS, Tumor Burden Score; CI, confidence interval.


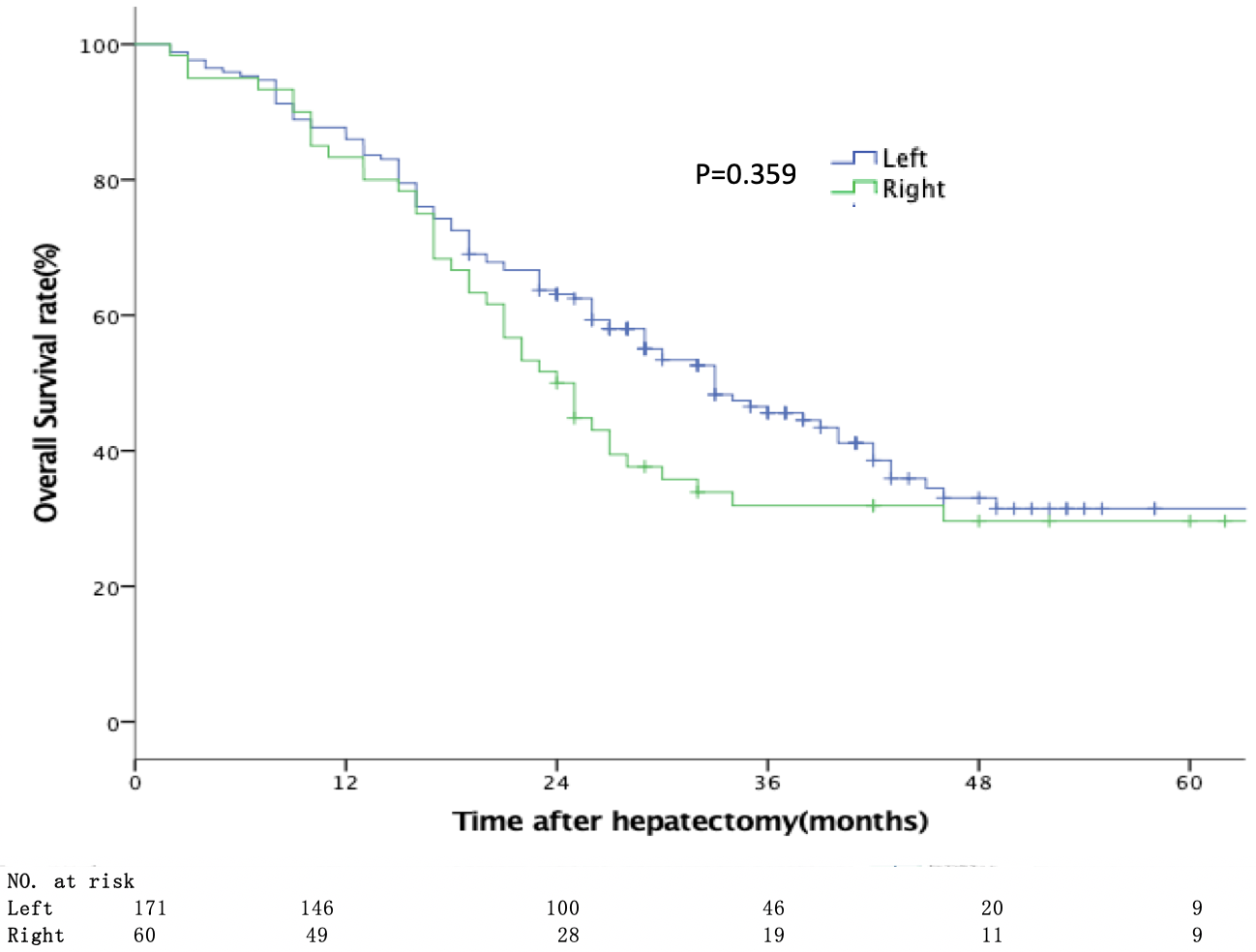


a

**
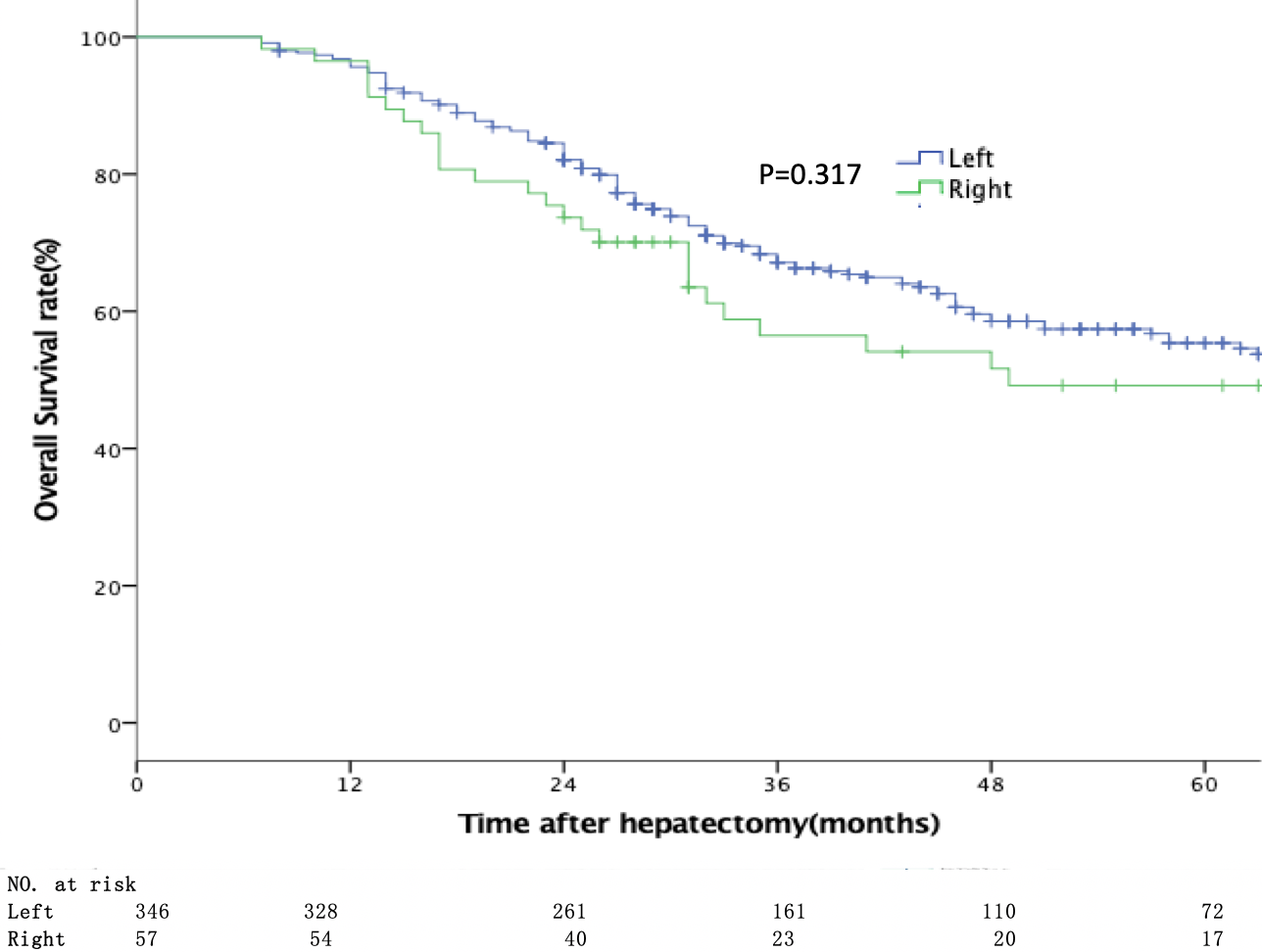
**

b

**Supplementary Figure1.** Overall survival after resection of colorectal liver metastases stratified by primary tumor location(Left versus Right). (a)In RAS/BRAF mutated-type patients. (b) In RAS/BRAF wild-type patients.
